# Biomass pretreatment: a critical choice for biomass utilization via biotechnological routes

**DOI:** 10.1186/1753-6561-8-S4-O34

**Published:** 2014-10-01

**Authors:** Ricardo SS Teixeira, Ayla S Silva, Rondinele O Moutta, Viridiana S Ferreira-Leitão, Rodrigo RO Barros, Maria Antonieta Ferrara, Elba PS Bon

**Affiliations:** 1 Departamento de Bioquímica, Instituto de Química, Universidade Federal do Rio de Janeiro, Centro de Tecnologia, Av. Athos da Silveira Ramos, 149 - Ilha do Fundão, Rio de Janeiro, RJ - CEP: 21941-909, Brazil; 2 Instituto Nacional de Tecnologia (INT), Ministério da Ciência e Tecnologia, Av. Venezuela, 82/302 - Rio de Janeiro, RJ - CEP: 20081-312, Brazil; 3 Instituto de Tecnologia em Fármacos - Far-Manguinhos/ FIOCRUZ Rua Sizenando Nabuco, 100 - Manguinhos 21041-250 - Rio de Janeiro - RJ, Brazil

## Introduction

The necessary biomass pretreatment step, to render the material accessible to the relevant enzyme pool, has been under thorough investigation as the production of biomass syrups, via enzymatic hydrolysis, with high sugars concentrations and yields and low inhibitors concentrations requires the pretreatment to be both efficient and low cost. A good choice for biomass pretreatment should be made by considering: (i) the possibility to use high biomass concentration; (ii) a highly digestible pretreated solid by either increasing the biomass superficial area or decrease in crystallinity or both; (iii) no significant sugar degradation into toxic compounds; (iv) yeast and bacterial fermentation compatibility of the derived sugar syrups; (v) lignin recovery; (vi) operation in reasonably sized and moderately priced reactors and (vii) minimum heat and power requirements [1].

Considering the most known pretreatments, such as diluted acid, hydrothermal processes, steam explosion, milling, extrusion, and ionic liquids, different pretreatment methods produce different effects on the biomass in terms of its structure and composition [2]. For example, the hydrothermal, steam explosion and acidic pretreatments conceptually remove mainly the biomass hemicellulose fraction whereas alkaline pretreatments remove lignin. On the other hand the product of a milling-based pretreatment retains the biomass initial composition. Furthermore, cellulose crystallinity is not significantly reduced by pretreatments based on steam, or hydrothermal, or acidic procedures, whereas ionic liquid-based techniques can shift crystalline cellulose into amorphous cellulose, substantially increasing the enzymatic hydrolysis rates and yields. As such, the choice of pretreatment and its operational conditions as well as the composition of the enzyme blend used in the hydrolysis step, determines the hexose and pentose sugars composition, the concentration and toxicity of the resulting biomass syrups. The activity profile of the enzyme blend and the enzyme load for an effective saccharification may also vary according to the pretreatment. Indeed, a low hemicellulase load can be used for a xylan-free biomass and a lower cellulase load will be needed for the hydrolysis of a low crystalline and highly amorphous pretreated biomass material.

As the pretreatment choice will also be affected by the type of biomass, the envisaged biorefinery model will need to consider the main types of biomass that will be used for the biorefinery operation so as to select an appropriate, and versatile pretreatment method [3]. Considering the biorrefinery concept which broadens the biomass derived products, the C6 sugars could be fermented into ethanol, while the C5 stream could be used for the production, via biotechnological routes, of a wide range of chemicals with higher added value. To date, sugarcane and woody biomass, depending on the geographic location, are strong candidates as the main renewable resources to be fed into a biorefinery. However, due to major differences regarding their physical properties and chemical composition, the relevant pretreatments to be used in each case are expected to be selective and customized. Moreover, a necessary conditioning step for wood size reduction, prior to the pretreatment, may not be necessary for sugarcane bagasse, affecting the pretreatment energy consumption and costs. Moreover, the choice of pretreatment should take into account the foreseen utilization of the main biomass molecular components (cellulose, hemicelluloses and lignin). It is important to point out that lignin can be used as a valuable solid fuel or as a source of aromatic structures for the chemical industry.

Sugarcane is one of the major agricultural crops when considering ethanol production, especially in tropical countries. In Brazil, sugarcane occupies 8.4 million hectares, which corresponds to 2.4% of farmable lands in Brazil. The gross revenue of this sector is about US$ 20 billion (54% as ethanol, 44% as sugar, and 2% as bioelectricity) [4]. In addition, up to 50% of all vehicles in Brazil are flex fuel cars, which corresponds to approximately 15 million cars [5]. Given the above, Brazil is an important player in this scenario, and, consequently, sugarcane bagasse and straw are promising feed stocks for biomass ethanol. Brazil produced, in 2008, 415 million tons of sugar cane residues, 195 million tons of sugarcane bagasse, and 220 million tons of sugarcane straw, whereas the forecast for the 2011 sugarcane production is 590 million tons, which would correspond to 178 million tons of bagasse, and 200 million tons of straw [6]. Currently, in Brazil, R&D on the use of biomass via biotechnological routes has been focused mainly on agricultural residues such as sugarcane residual biomass.

## Advantages and disadvantages of different types of pretreatments

**Acid pretreatment**. Pretreatment with dilute sulfuric acid has been reported as one of the most widely used processes due to its high efficiency. This pretreatment removes and hydrolyzes up to 90% of the hemicellulose fraction, rendering the cellulose fraction more accessible to hydrolytic enzymes. However, it presents important drawbacks related to the need for a neutralization step that generates salt and biomass sugar degradation with the formation of inhibitors for the subsequent fermentation step such as furfural from xylose degradation. The removal of inhibitors from the biomass sugar syrups adds cost to the process and generates a waste stream. Additionally, mineral acids are corrosive to the equipment, calling for the use of more sturdy materials alongside higher maintenance costs. Acid recovery is also costly. The availability of the biomass acid pretreatment and the knowledge that has been built up on this subject highlights its important and costly drawbacks. In addition, the environmental problems caused by its waste streams have called for the need for other options for the pretreatment of lignocellulosic materials.

**Mechanical pretreatments**. Mechanical pretreatments of biomass aim primarily to increase the surface area by reducing the feedstock particle size, combined with defibrilization or reduction in the crystallinity degree. This approach facilitates the accessibility of enzymes to the substrate, increasing saccharification rates and yields. The most studied biomass mechanical pretreatment for biomass is the milling process, mainly the ball-milling, which presents a high energy consumption, and wet disk-milling pretreatments [7,8]. Another mechanical treatment to be considered is extrusion, even though this process involves additional thermal and/or chemical pretreatments.

**Liquid hot water (LHW) pretreatments**. The liquid hot water (LHW) is based on the use of pressure to keep water in the liquid state at elevated temperatures (160-240 ºC). This process changes the biomass native structure by the removal of its hemicellulose content alongside transformations of the lignin structure, which make the cellulose more accessible to the further enzymatic hydrolysis step. Differently from steam-explosion treatment, LHW does not use rapid decompression and does not employ catalysts or chemicals. Nevertheless, as with the acid treatment, LHW depolymerizes hemicelluloses to the liquid fraction. In this case, sugars are removed mostly as oligosaccharides, and the formation of the inhibitors furfural and 5-hydroxymethyfurfural (HMF) is at a slightly lower level, depending on the process conditions. To avoid the formation of inhibitors, the pH should be kept at between 4 and 7 during the pretreatment, because at this pH, hemicellulosic sugars are retained in oligomeric form, and monomer formation is minimized. The removal of hemicellulose also results in the formation of acetic acid in the liquid fraction. LHW and steam pretreatments are attractive from a cost-savings perspective, as they do not require the addition of chemicals such as sulfuric acid, lime, ammonia, or other catalysts. Moreover, the reactors do not require high cost materials and maintenance due to their low-corrosion potential. Additionally, these treatments do not alter the biomass glucan content, as a glucose recovery rate of 97% was observed for sugarcane bagasse that was pretreated by both methods. The main differences between the features of the two treatments relates to hemicellulose extraction, which is higher for the LHW, and the biomass load, which is higher for the steam pretreatment, with the obvious corresponding advantages and disadvantages. In contrast to steam pretreatment, LHW allows for a higher pentosan recovery associated with the lower formation rate of inhibitors.

**Steam-explosion pretreatment**. The main advantages of steam explosion relate to the possibility of using coarse particles, thus avoiding a biomass-size conditioning step, the non-requirement for exogenous acid addition (except for softwoods, which have a low acetyl group content in the hemicellulosic portion), a high recovery of sugars, and the feasibility for industrial implementation. Moreover, the soluble stream rich in carbohydrates derived from hemicellulose in the form of oligomers and monomers may be easily removed and used as feedstock for the production of higher added-value products such as enzymes and xylitol. Other attractive features include less hazardous process chemicals and conditions, the potential for significantly lower environmental impact, and lower capital investment. The fact that the steam-explosion process does not require previous grinding of the raw biomass is an important feature, considering that the energy required to reduce the particle size before the pretreatment (pre-grinding) can represent up to one-third of the total energy required in the process. The main drawbacks related to steam-explosion pretreatment are the enzyme and yeast inhibitors generated during the pretreatment, which include furfural and hydroxymethyl furfural; the formation of weak acids, mostly acetic, formic, and levulinic acids, the two latter acids being derived from furfural's and hydroxymethyl furfural's further degradation; and the wide range of phenolic compounds produced due to lignin breakdown. Several detoxification methods have been developed in order to reduce the inhibitory effect, which represent additional costs in the overall process. Other limitations of this method include the incomplete disruption of the lignin-carbohydrate matrix.

**Ionic liquids pretreatment**. ILs are able to disrupt the plant cell wall structure by the solubilization of its main components. This class of salts is also able to alter cellulose crystallinity and structure, rendering the amorphous cellulose prone to high rates and yields from enzymatic saccharification. Indeed, this combination of effects generates a pretreated material that can be easily hydrolyzed into monomeric sugars when compared to other pretreatment technologies, also rendering the enzymatic attack faster as the initial hydrolysis rate is greatly increased [9,10]. Nevertheless ILs are still too expensive to be used for biomass pretreatment at the industrial scale, as a inovative and promising biomass pretreatment technologies, the use of IL stands out. These versatile classes of chemicals can be tailored to suit the selective extraction and recovery of the biomass components, such as the recovery of a cellulose-hemicellulose rich material in an amorphous form which is prone to enzymatic hydrolysis with high yields and rates. Additionally, the possibility of recovering the extracted lignin broadens and increases the efficiency for the use of biomass.

**Alkaline pretreatment**. In the alkaline process the biomass is soaked in the alkaline solution and mixed at a mild controlled temperature in a reaction time frame from hours to days. It causes less sugar degradation than the acidic pretreatments. The necessary neutralizing step, prior to the enzymatic hydrolysis, generates salts that can be partially incorporated to the biomass. Besides removing lignin the pretreated material washing also removes inhibitors, salts, furfural and phenolic acids. This pretreatment, whereby sodium hydroxide has been the most studied reagent is similar to the Kraft pulping process used in the pulp and paper industries. The main effect of alkaline pretreatments is the biomass lignin removal thereby reducing the steric hindrance of hydrolytic enzymes and improving the reactivity of polysaccharides. The addition of air/oxygen to the reaction mixture dramatically improves delignification. The alkali pretreatment also causes partial hemicellulose removal, cellulose swelling and cellulose partial decrystallization.

## Conclusion

Several factors must be taken into account regarding the choice for biomass pretreatment regarding the most advantageous use of the biomass solid and liquid streams resulting from the subsequent enzymatic hydrolysis step. The resulting sugar syrups stream and the lignin stream, as either a solid or a liquid form must be carefully considered for the deployment of a fully integrated biorefinery, for the use of biomass as a source of fuels and chemicals in a sustainable an environmentally friendly way.
